# Clinical characteristics and 6-month follow-up of adults with and without alcohol use disorder who self-harm

**DOI:** 10.3389/fpsyt.2024.1396855

**Published:** 2024-08-02

**Authors:** Petter Olsson, Stefan Wiktorsson, Lotta M. J. Strömsten, Ellinor Salander Renberg, Bo Runeson, Margda Waern

**Affiliations:** ^1^ Department of Psychiatry and Neurochemistry, University of Gothenburg, Gothenburg, Sweden; ^2^ Region Västra Götaland, Sahlgrenska University Hospital, Psychosis Clinic, Mölndal, Sweden; ^3^ Department of Psychology, Umeå University, Umeå, Sweden; ^4^ Department of Clinical Sciences, Psychiatry, Umeå University, Umeå, Sweden; ^5^ Department of Clinical Neuroscience, Centre for Psychiatry Research, Karolinska Institute and Stockholm Health Care Services, Stockholm, Sweden

**Keywords:** suicide, alcohol, self-harm, suicidal behavior, suicide risk

## Abstract

**Background:**

Alcohol use disorder (AUD) is associated with suicidal behavior, but prospective clinical studies are lacking.

**Aim:**

To compare clinical characteristics and 6-month outcomes in persons with and without AUD who self-harm.

**Methods:**

804 adults (mean age 33, age range 18-95, 541 women and 263 men, 666 with suicide attempts and 138 with non-suicidal self-injuries at index) at three Swedish university hospitals took part in a research interview that included the Mini International Neuropsychiatric Interview (MINI). Subsequent non-fatal suicidal behavior within six months was identified by record review; suicides were identified by national register.

**Results:**

At index, 39% of the men and 29% of the women had AUD. Over two thirds of these cases (69%) were identified by the MINI, but not by clinical AUD diagnosis. While trait impulsivity was more common among persons with AUD than those without (56% vs 36%, *P _adj_ =* <.001), impulsivity in connection with the index attempt was noted in half of the participants in each group (48% vs 52%, *P _adj_ =* 1). Subsequent suicidal behavior (fatal/non-fatal) occurred in 67 persons with AUD (26%) and in 98 without AUD (18%), a 60% higher risk among persons with AUD (OR = 1.60, 95% [*CI* 1.13-2.28], *P = .*009). Four persons with AUD (2%) and six without (1%) died by suicide within 6 months.

**Conclusion:**

Almost a third of patients presenting at psychiatric emergency settings after self-harm fulfilled criteria for AUD, but clinicians often missed this diagnosis. Risk for subsequent suicidal behavior was elevated in patients with AUD. Educational interventions to improve recognition of alcohol use disorder may aid clinicians in the assessment and management of patients who present with self-harm.

## Introduction

Alcohol use disorder (AUD) is an important risk factor for suicidal behavior (1, 2). The prevalence of AUD is estimated at around 1.4% worldwide, with a 70/30 male-to-female ratio (3). It is also more common in psychiatric populations (4). In a recent meta-analysis, alcohol use was associated with a 94% increase in risk of death by suicide (5). While findings diverge regarding severity of AUD and prospective risk of suicide (6, 7), other clinical characteristics such as impulsivity likely contribute to higher risk (8, 9). In general populations, it has been suggested that the disruptive effects of alcohol use on day-to-day functioning (often measured using the Alcohol Use Disorders Identification Test, AUDIT) may play a greater role in the relationship between alcohol use and suicide-related outcomes than higher alcohol consumption (10). AUD is also a risk factor for attempted suicide (11), even in later life (12). In a study by Haw et al., 27% of persons with an episode of deliberate self-harm were dependent on alcohol or used it harmfully (13). Other reports have shown that persons with combined alcohol misuse and self-harm are at increased long-term risk of repeated self-harm and of suicide compared to those who self-harm but do not misuse alcohol (14, 15). Frequently, persons with AUD and self-harm present with additional psychiatric comorbidities, with depression being the most prevalent (16).

Although research has shown a prospective relationship between AUD and suicidal behavior (17–20), we were able to identify only a few studies investigating the prospective short-term relationship between alcohol problems and suicidal behavior in adults who self-harm (21). In the first study, which was set in France, alcohol addiction was associated with repeated deliberate self-harm within 6 months of a deliberate self-harm episode. The study only included self-poisonings and the sample was relatively small. The second study examined the prospective association between AUD and deliberate self-harm and suicide by any method in a large sample (22). No increase in suicide was found among those with AUD and self-harm.

Suicide risk is known to be particularly elevated shortly following discharge from psychiatric hospital admission, and short-term prospective studies investigating repeat suicidal behavior in this critical time frame have been specifically requested (23). The aim of this multicentre study was to compare clinical characteristics in persons with and without AUD who present with self-harm including short-term prospective risk of suicidal behavior.

## Materials and methods

### Participants

Eight hundred and four patients (541 women, 263 men) aged 18 and older were recruited and interviewed at three university hospitals in Sweden [Sahlgrenska University Hospital in Gothenburg (*n* = 190), St Göran’s Hospital, Karolinska Institute, Stockholm (*n* = 479) and the Norrland University Hospital in Umeå (*n* = 135)] in connection with a suicide attempt or an episode of non-suicidal self-injury (NSSI). Recruitment was carried out between April 2012 and March 2016. Inability to complete the interview due to insufficient language skills, cognitive dysfunction, severe psychosis, delirium, dementia or severe somatic conditions resulted in exclusion. Total participation rate was 71% (24). The median age of participants was 33 years (25^th^ percentile = 23, 75^th^ percentile = 50, standard deviation (*SD)* 18.1, range 18 – 95) (25). Women were younger than men (30 vs 38, Mann-Whitney *U =* 58110.5, *z-*score = -4.22, *P-*value (*P*) *<*.001) (26).

At baseline, 666 participants (83% of the total sample) presented with a suicide attempt, defined in accordance with the Columbia Suicide Severity Rating Scale (C-SSRS) (27) as an act of physical self-injury with at least some degree of intent to die. In the remaining 138 participants (17%), the self-injury was completely without intent to die and was thus defined as non-suicidal self-injury (NSSI). Methods employed at the index self-harm episode are shown for each behavior type in **Supplementary Table 1**.

### Procedure

Interviews were conducted by mental health staff trained in the application of the assessment instruments (psychiatrists, psychiatric nurses, psychologists and psychiatric trainees) within a mean of 3.9 (median = 3) days after emergency psychiatric admission. Interview duration averaged 1-2 hours.

### Diagnostics and instruments

Clinical diagnoses at the index episode were retrieved from psychiatric records. In addition, the *Mini International Neuropsychiatric Interview (MINI)* (28) was included in the research interview to provide standardized diagnostics across study centers. The MINI is a structured interview instrument, designed to give a brief and accurate assessment of psychiatric disorders. For the purpose of this study, a person was considered to have AUD if they had received an International Statistical Classification of Diseases (ICD-10) diagnosis of F10.1 (harmful use of alcohol) or F10.2X (alcohol dependence) in connection with the index episode, or if they qualified for past year alcohol dependence or abuse in accordance with the MINI.

Rating scales employed in this study are presented below; further details are provided in **Supplementary Table 3**.

The *Columbia Suicide Severity Rating Scale (C-SSRS)* (27) was used to define suicide attempts or NSSI and to determine medical lethality of the index episode. Lethality was rated according to the C-SSRS item 21a (actual lethality/medical harm of most recent suicide attempt) on a scale of 0-5. For the purpose of this study, a rating ≥3 was used to define high lethality.

The *Suicide Assessment Scale (SUAS)* (29) was included in the interview to measure symptoms of suicidality, regardless of diagnosis. Items are rated on a scale of 0-4, with higher values indicating greater symptom severity. In the current study we applied the items denoting feelings of hopelessness and worthlessness, worries and trait impulsivity. A rating ≥2 was used to define high severity in each of these items.

The *Suicide Intent Scale (SIS)* (30) is designed to measure degree of suicidal intent. Items are rated on a scale of 0-2. In our study, impulsivity in connection with the actual index attempt was rated on a scale of 0-2 according to item 15 (degree of preparation). In this study, a score of 0 (*no preparations, impulsive act*) defined the attempt as impulsive.

The *Alcohol Use Disorders Identification Test (AUDIT)* (31) is a questionnaire developed to evaluate extent of alcohol use and problems related to alcohol use. It contains 10 questions scored between 0-4 (total range 0-40) with higher scores denoting higher consumption/more severe alcohol related problems. In accordance with WHO guidelines (32), we set the cut-off score to indicate hazardous use at 8 for men aged <65 and 7 for all women and for men aged >65.

The *Karolinska Affective and Borderline Symptoms Scale, Self-report (KABOSS)* (33) was administered to capture symptoms of common mental disorders as well as symptoms related to emotionally unstable personality disorder (EUPD). KABOSS includes the *Comprehensive Psychopathological Rating Scale for Affective Syndromes (CPRS-S-A)*, a brief self-rated scale commonly used in psychiatric clinics in Sweden. The nine-item depression subscale included herein is the internationally well-known *Montgomery-Åsberg Depression Self-Rating Scale (MADRS-S)* (34) and the nine-item subscale for the rating of anxiety symptoms is the *Brief Scale for Anxiety (BSA)* (35). The KABOSS also includes eight items corresponding to diagnostic DSM-V criteria for EUPD (mood swings, ability to understand one’s own feelings, self-control, ability to comfort oneself, feelings of abandonment, emptiness, self-image, and reality presence). All KABOSS items are scored 0-6, with higher numbers denoting higher severity. For the item assessing feelings of abandonment, a rating of ≥2 was set to define presence of such feelings.

As previously described (24), interrater agreement was excellent for the interviewer-rated instruments SIS, C-SSRS and SUAS (intraclass correlation, ICC = 0.99, *P* <.001).

### Outcomes

The main outcome for this study was subsequent suicide attempts (fatal or non-fatal) within 6 months of the index episode. Non-fatal suicide attempts (with any degree of suicide intent) as well as employed methods were recorded during review of in- and outpatient records. Cutting, hanging, gassing, drowning, jumping from heights, shooting and methods involving vehicles were classified as violent methods. The Swedish National Cause of Death Register was used to identify suicides that occurred during the 6-month follow-up. The unique Swedish personal number allowed linkage of register data to research interview and medical record data.

### Statistics

For SUAS and KABOSS, missing data was tested for missingness pattern using Little’s Missing Completely at Random (MCAR) test. In accordance with recommendations by Scheffer (36), assessment scale values identified as MCAR or missing at random (MAR) were imputed by single imputation using the expectation maximum algorithm within the statistical software SPSS^®^. For SUAS, data was treated as MAR instead of MCAR, Little’s MCAR test; *χ^2^* (246, n = 804) = 1372, *P* = .01. When case data was partially missing (n = 38), in total 5% of SUAS data, values were imputed. Where no data was imputed, cases with missing data were excluded from the present analysis. For KABOSS, data was also treated as MAR, Little’s MCAR test; *χ^2^* (888, n = 804) = 988.58, *P* = .01. For cases with less than 25% missing data (n = 45), values were imputed by single imputation. Cases with 25-100% missing data (n = 133) were excluded listwise. Deviations from normal distribution were identified through inspection of histograms. *T*-tests were used for continuous variables, to investigate group differences between patients with and without AUD. Proportions were compared using Chi-square tests. The Mann-Whitney U Test was used for age comparisons since age was not normally distributed. We employed binary logistic regression models to calculate odds ratios for new fatal or non-fatal suicide attempts and analyses were adjusted for age, sex and illicit drug use. Two separate regression models were created to calculate odds ratios for recently increased alcohol consumption- and relationship problems- prior to index episode, respectively. All *t*-tests yielded similar results when cross-checked with non-parametric equivalents (Mann-Whitney U Test). A Bonferroni correction for multiple analyses was conducted for **Tables 1**, **2**, where 45 total tests yielded adjusted P-values. All statistical analyses were performed using SPSS^®^ version 24 for Windows.

**Table 1:** Sociodemographic and clinical characteristics at index self-harm episode, by AUD status (N=804).

	AUD, n = 258 n (%)	No AUD, n = 546 n (%)	*χ^2^*	*df*	*P*	*P_adj_* ^a^	Missing data n^b^
**Women**	155 (60)	386 (71)	9.0	1	**.003**	.135, *ns*	**-**
Employment							
Student	36 (14)	92 (17)	1.1	1	.295, *ns*	1, *ns*	–
Working	109 (42)	172 (32)	8.9	1	**.003**	.135, *ns*	**-**
Unemployed	52 (20)	82 (15)	3.3	1	.068, *ns*	1, *ns*	–
Retired	13 (5)	71 (13)	11.9	1	**<.001**	**<.001**	**-**
Sick leave	59 (23)	155 (28)	2.7	1	.098, *ns*	1, *ns*	–
Disability pension	21 (8)	62 (11)	2.0	1	.162, *ns*	1, *ns*	–
Marital status							
Never married/cohabiting	118 (46)	226 (41)		1	.245, *ns*	1, *ns*	–
Currently married/cohabiting	68 (27)	136 (25)		1	.660, *ns*	1, *ns*	–
Divorced/separated	67 (26)	151 (28)		1	.616, *ns*	1, *ns*	–
Widow/Widower	3 (1)	33 (6)		1	**.002**	.090, *ns*	–
Clinical diagnosis at baseline^c^							
Dementia (F00-09)	3 (1)	5 (1)	0.1	1	.742, *ns*	1, *ns*	–
Drug misuse (F11-16, F18-F19)^d^	47 (18)	43 (8)	18.9	1	**<.001**	**<.001**	**-**
Psychosis (F20-29)	6 (2)	20 (4)	1.0	1	.317, *ns*	1, *ns*	–
Mood disorder (F30-39)	75 (29)	220 (40)	9.5	1	**.002**	.090, *ns*	**-**
Anxiety, stress (F40-48)	91 (35)	229 (42)	3.3	1	.071, *ns*	1, *ns*	–
Eating disorder (F50-59)	3 (1)	9 (2)	0.3	1	.596, *ns*	1, *ns*	–
Emotionally unstable personality disorder (F60.3)	43 (17)	94 (17)	0.04	1	.847, *ns*	1, *ns*	–
Developmental disorder (F70-79)	3 (1)	12 (2)	1.0	1	.311, *ns*	1, *ns*	–
Autism spectrum disorder (F80-89)	13 (5)	41 (8)	1.7	1	.191, *ns*	1, *ns*	–
High medical lethality, index attempt C-SSRS 21a	88 (38)	148 (31)	4.2	1	.055, *ns*	1, *ns*	87
Impulsivity during actual index attempt, SIS 15	122 (48)	276 (52)	5.6	1	.235, *ns*	1, *ns*	19
Worries/anxiety SUAS 9	225 (87)	438 (80)	5.9	1	**.015**	.645, *ns*	–
Trait impulsivity, SUAS 11	144 (56)	198 (36)	27.4	1	**<.001**	**<.001**	–
Worthlessness SUAS 12	226 (88)	452 (83)	3.1	1	.080, *ns*	1, *ns*	–
Hopelessness SUAS 13	218 (85)	443 (81)	1.4	1	.245, *ns*	1, *ns*	–
Feelings of abandonment KABOSS	190 (74)	345 (63)	8.6	1	**.003**	.135, *ns*	–
Increased alcohol use prior to index episode	104 (42)	45 (9)	116	1	**<.001**	**<.001**	44
Alcohol was used at index attempt^e^	153 (71)	129 (30)	13.6	1	**<.001**	**<.001**	13
Recent relationship problems	186 (72)	340 (62)	7.3	1	**.007**	.315, *ns*	1
Scales	Mean score		*T*	*df*	*P*		
Alcohol use (AUDIT)	17.9	4.9	22.8	575	**<.001**	**<.001**	227
Depression (MADRS)	28.5	27.4	1.2	669	.233, *ns*	1, *ns*	158
Anxiety (BSA)	22.9	21.4	1.9	669	.054, *ns*	1, *ns*	152
Borderline symptoms (KABOSS)	28.8	25.7	3.0	650	**.002**	.090*, ns*	133

AUD Alcohol use disorder, C-SSRS 21a Columbia- Suicide Severity Rating Scale item 21a, SIS 15 Suicide Intent Scale item 15, SUAS 9/11/12/13 Suicide Assessment Scale item 9/11/12/13, KABOSS Karolinska Affektiva och Borderline Symptoms Scale, AUDIT Alcohol Use Disorders Identification Test, MADRS Montgomery-Åsberg Depression Rating Scale, BSA Brief Scale for Anxiety. ^a^P-values adjusted for multiple analyses (Bonferroni correction, total tests n = 45) ^b^No significant differences in proportions missing between groups ^c^Participants could have more than one diagnosis. ^d^Alcohol and tobacco diagnoses excluded. ^e^Only actual suicide attempts according to C-SSRS (n = 666).

Bold values indicate statistical significance at the 0.05 level.

**Table 2:** Follow-up care during 6 months after index episode of suicide attempt or non-suicidal self-injury.

	AUD, n = 258 n (%)	No AUD, n = 546 n (%)	*χ^2^*	*df*	*P*	*P_adj_* ^a^	Missing data n^b^
Type of care							
Referral to psychiatric outpatient care	195 (76)	473 (87)	16.8	1	**<.001**	**<.001**	3
Recorded visit to psychiatric outpatient care	177 (71)	438 (81)	10.1	1	<.**001**	**<.001**	10
Recorded visit psychiatric emergency services	104 (41)	217 (40)	0.11	1	.74*, ns*	1*, ns*	7
Referral to treatment for substance abuse	102 (40)	44 (8)	115	1	**<.001**	**<.001**	4
Recorded visit to treatment for substance abuse	103 (41)	41 (8)	129	1	**<.001**	**<.001**	10
Referral to primary care	38 (15)	116 (21)	5.01	1	.**025**	1*, ns*	4
Psychological treatment	104 (40)	245 (45)	1.65	1	.20*, ns*	1*, ns*	3
Pharmacological treatment							
Antidepressants	178 (70)	404 (75)	2.89	1	.09*, ns*	1*, ns*	11
Mood stabilizers	50 (20)	86 (16)	1.29	1	.26*, ns*	1*, ns*	19
Antipsychotics	108 (42)	195 (37)	2.00	1	.16*, ns*	1*, ns*	17
Anxiolytics	218 (85)	448 (84)	0.03	1	.87*, ns*	1*, ns*	16
ADHD medication	21 (8)	46 (9)	0.05	1	.83*, ns*	1*, ns*	17

AUD Alcohol use disorder, ADHD Attention Deficit and Hyperactivity Disorder.

a P-values adjusted for multiple analyses (Bonferroni correction, total tests n = 45).

b No significant differences in proportions missing between groups.

Bold values indicate statistical significance at the 0.05 level.

### Ethics statement

The authors assert that all procedures contributing to this work comply with the ethical standards of the relevant national and institutional committees on human experimentation and with the Helsinki Declaration of 1975, as revised in 2008. All procedures involving human subjects/patients were approved by the Regional Ethics Committee in Gothenburg, (589-10, T034-12). All participants were fully informed of the study proceedings and gave written consent.

## Results

A clinical diagnosis of AUD was noted in the medical records in connection with the index episode for 80 participants (10%). An additional 178 persons fulfilled criteria for past year AUD in accordance with the MINI, yielding a total of 258 persons with AUD (32% of the total cohort). This diagnosis was more common among men (40%), but over a fourth of the women (29%) also had AUD (*χ^2 ^=*8.98, *P =* 0.003).

### Baseline characteristics in persons with and without alcohol use disorder

Median age was numerically lower among persons with AUD (Mdn = 31.5) than in those without (Mdn = 33.5), although the difference was not statistically significant (*z* = -1.94, *P* <.052). Sociodemographic and clinical characteristics of study participants with and without AUD are presented in **Table 1**. Trait impulsivity and illicit drug use were more common among persons with AUD compared to people without AUD. Mood disorders were less common in the AUD group than in the non-AUD group (29% vs 40%, *p* = 0.002), although this finding did not survive correction for multiple analyses. Proportions of other clinical diagnoses were similar in both groups. Regarding proportions presenting with NSSI at the index episode, no group differences were found. Neither MADRS nor Brief Scale for Anxiety scores differed in those with and without AUD. Proportions with high medical lethality in the index attempt did not differ. While trait impulsivity was more common among persons with AUD, proportions with impulsivity in connection with the actual index attempt were the same (about 50%) in participants with and without this condition.

Mean AUDIT scores were higher in persons with AUD (17.9 vs 4.9, *P* <.001). Within the AUD group, mean AUDIT scores were higher in those with a clinical diagnosis of AUD (mean 21.2, range 0-40) than in those who were diagnosed with MINI only (mean 16.6, range 0-39) (*P = .*001). However, proportions scoring above the WHO threshold for hazardous use were similar (92% in the group with a clinically diagnosed AUD vs 90% in the group identified by the MINI only, *χ^2 ^=*0.76, *P =* 0.78).

### Treatment during follow-up

**Table 2** details the distribution of follow-up care during the 6-month observation period. Persons with AUD were more likely to receive a referral to- or to have at least one recorded visit to treatment for substance use disorder. They were less likely to be referred to psychiatric outpatient care and primary care, and also less likely to have a recorded visit to psychiatric outpatient care in the aftermath of the index episode. There were no significant differences in distribution of pharmacological treatments between groups.

### Suicidal behavior during follow-up

For the total group, the number of persons with at least one episode of fatal- or non-fatal suicidal behavior within 6 months was 165 (20.5%). These events were registered in 67 persons with AUD (26.0%) and in 98 without (17.9%). Among these, four persons with AUD (2%) and six without this diagnosis (1%) died by suicide. Of the 165 participants with subsequent suicidal behavior, 55 (33.3%) used a violent method (**Supplementary Table 1**), with no difference in proportions between patients with and without AUD (26.9% vs 37.8%, *P = .*145).

### Regression models

**Table 3** details binary regression models for predicting the main study outcome (new fatal or non-fatal suicide attempts within 6 months). Other models are detailed in **Supplementary Table 2**. In an unadjusted binary logistic regression model, persons with AUD were 60% more likely to have at least one episode of suicidal behavior during follow-up (OR = 1.60, [95% *CI* 1.13-2.28], *P = .*009). The results remained significant in a model adjusting for illicit drug use, sex and age (OR = 1.49, [95% *CI* 1.04-2.15], *P = .*03). Illicit drug use was not an independent predictor in this model (*P = .*33). In unadjusted regression models, neither total AUDIT score (OR = 1.01, [95% *CI* 0.99-1.03], *P = .*49) nor a rating above the WHO AUDIT cut-off for hazardous alcohol use (OR = 0.97, [95% *CI* 0.66-1.44], *P =* 0.89) predicted subsequent suicidal behavior.

**Table 3:** Logistic regression models, prediction of new suicide attempts (fatal and non-fatal) within 6 months.

	Model			Predictor						
	Nagelkerke *R^2^*	*χ^2^*	*P*	B	SE	Wald	*df*	*P*	Exp(B)	95% CI
AUD, unadjusted	.013	6.71	**.01**	0.47	0.18	6.84	1	**.01**	1.60	1.13-2.28
AUD + substance misuse + sex + age	.041	21.07	**<.001**							
AUD				0.40	0.19	4.63	1	**.03**	1.49	1.04-2.15
Substance misuse				0.26	0.27	0.97	1	.33, *ns*	1.30	0.77-2.18
Sex				0.08	0.20	0.17	1	.68, *ns*	1.09	0.74-1.59
Age				-0.02	0.01	11.15	1	**.001**	0.98	0.97-0.99
AUDIT total score, unadjusted	.001	0.47	.49, ns	0.01	0.01	0.48	1	0.49, *ns*	1.01	0.99-1.03
AUDIT WHO cut-off, unadjusted	<.001	0.02	.89, ns	-0.03	0.20	0.02	1	0.89, *ns*	0.97	0.66-1.44

AUD Alcohol Use Disorder, AUDIT Alcohol Use Disorders Identification Test, WHO World Health Organization.

Bold values indicate statistical significance at the 0.05 level.

In binary logistic regression models adjusted for age and sex, those with AUD were more than seven times as likely to report recently increased alcohol consumption prior to the index episode (OR = 7.16, [95% *CI* 4.80-10.69], *P <*.001), and more than 50% more likely to have experienced relationship problems before the index episode (OR = 1.55, [95% *CI* 1.11-2.16], *P = .*010). Among those with an actual suicide attempt at baseline, persons with AUD were more than five times as likely to have used alcohol in connection with the attempt (OR = 5.97, [95% *CI* 4.14-8.59], *P <*.001). However, reporting increased alcohol consumption prior to index SA/NSSI did not have an impact on the short-term risk of future suicidal behavior (OR = 0.99, [95% *CI* 0.64-1.55], *P = .*98).

## Discussion

Almost a third of the participants in our self-harm clinical cohort fulfilled criteria for AUD. However, this diagnosis was lacking in the medical notes for two thirds of the cases identified by the MINI neuropsychiatric interview. This finding highlights the need for improved recognition of AUD in the context of hospital presentations for self-harm. AUD was associated with increased likelihood of fatal/non-fatal suicidal behavior within six months of the index self-harm episode. While most previous studies have focused on the long-term effect of AUD on repeat suicidal behavior, our findings suggest that persons with AUD may be especially exposed to short-term risk of new suicide attempts following self-harm.

Almost three quarters of the participants with AUD in our study had experienced recent interpersonal loss prior to the index self-harm episode, and this figure was significantly lower among those without AUD in the unadjusted analysis. Relationship problems have been suggested to play a key role in increasing risk of suicide among persons with AUD (37). The high prevalence of recent loss in those with AUD in our study expands on previous research focusing on suicide death (38), suggesting a core vulnerability of this group. Social connections should be thoroughly investigated in persons with AUD who self-harm. The connection between AUD, interpersonal loss and suicidal behavior is likely complex. While alcohol problems (and suicidal behavior) may be triggered by relationship collapse, it may also be a factor destabilizing those same relationships. Further research is needed on how these elements interplay.

Proportions with diagnosed emotionally unstable personality disorder (EUPD) were similar in those with and without AUD but trait impulsivity was more common in persons with AUD. The latter is of clinical relevance as having an impulsive personality has previously been linked to increased suicide risk in person with AUD (8, 9). Even though participants with AUD were more often characterized by the impulsivity trait, it should be noted that impulsivity in connection with the index attempt was noted in half of the participants both with and without AUD.

Although comorbid illicit drug use was more than twice as common among persons with AUD, persons with both diagnoses did not have an elevated risk of future suicidal behavior compared to those with AUD only. This was somewhat unexpected considering previous studies demonstrating that comorbid AUD confers additional risk of suicide attempts in persons with illicit drug use (39). While this could reflect a power issue, it must be pointed out that all participants in our study could be seen as a group with elevated risk as all had at least one episode of self-harm. Also, since persons with AUD were more likely to receive treatment for substance abuse, this additional level of care may also result in extra protection against new suicidal behavior.

The finding that less than a third of participants with AUD identified by the MINI had not received a clinical diagnosis is of relevance for patient safety, especially considering the increased risk for future suicidal behavior noted in the current study. Neither AUDIT score nor a score indicating hazardous drinking were associated with subsequent suicidal behavior in our study. It should be noted, however, that many persons had missing AUDIT scores which weakened power for these analyses. Experiencing a relapse to problematic levels of drinking has been considered a trigger for suicidal behavior, and persons with AUD are indeed more at risk of suicide during periods of active drinking (40). In this study, using alcohol in connection with the index attempt was very common, and significantly more common among persons with AUD. The finding parallels previous reports focusing on suicide death, where positive blood alcohol concentrations have been found in 23-69% of suicide decedents (41, 42). Persons with AUD were also much more likely to report increased alcohol consumption prior to the index attempt (40), although reporting so did not affect odds of new suicide attempt within 6 months.

“Deaths of despair” is a term used to describe premature deaths from suicide, alcohol use disorders and drug use/overdoses. Originally coined by economists Case and Deaton (43), the term has been a topic of interest in recent years, especially in the US where this phenomenon has been hypothesized to play a major role in rising mortality rates among adults. Copeland et al. (44) suggested a range of symptoms of despair including hopelessness, loneliness, worthlessness, helplessness, worries, self-pity and feeling unloved, and demonstrated that these cognitive indicators of despair were associated with suicidal thoughts and behaviors in young adults. In our study, which included adults of all ages, worries and feelings of abandonment characterized larger proportions of those with AUD compared to those without this diagnosis. While these findings did not survive the Bonferroni correction, they do suggest a need for further research. These phenomena might prove important in needs assessment for suicidal persons with AUD. The concept of deaths of despair has been little investigated to date in Swedish settings, but international research has indicated that certain high-income countries may provide some protection against despair, for example through strong social support (45). As economic and political tensions rise globally, however, deaths of despair in European settings may become a relevant topic for future research.

### Strengths and limitations

The baseline interview protocol for this study was comprehensive, allowing thorough characterization of patients with and without AUD. All interviews were carried out by psychiatric health professionals. and included objective- as well as self-rated measures. The prate was very good (>70%). Diagnoses were retrieved through review of psychiatric records. For AUD, we also included persons who qualified for the diagnosis on the MINI. Substance use disorders are somewhat notorious for being missed by clinicians (46), and adding another instrument for diagnosing AUD allowed for inclusion of those not identified in case records. It may be argued that the MINI may be generous in diagnosing AUD, especially since those clinically diagnosed with AUD had higher mean scores on the AUDIT than those who were diagnosed with MINI. However, scoring very high on the AUDIT was equally common in both groups.

It is known that persons with a recent admission to psychiatric inpatient care are at higher risk of suicide (47), and previous studies have pointed out the need to improve prediction of suicide in shorter time intervals (23). The short follow-up time of six months used in this study enhances relevance in the clinical setting and allows results to be more readily contextualized. However, several limitations require consideration. Participants were interviewed at baseline only and outcomes were based on medical record review and the National Cause of Death Register, which means that persons who survived suicide attempts but did not seek medical care are missed by our study design. Exposure variables were assessed at one time point only; we lack data on psychopathology and life events during the 6-month follow-up period. No data was available on prescription of drugs for treating AUD. Further, as is often the case with prospective clinical studies, we lacked power to carry out separate analyses to estimate risk of suicide death. Missing scores for certain subscales also affected power in a few models. Some results that were significant at the 0.05 level were no longer significant after correcting for multiple analyses. While it is useful to be aware of the risk of false positives, there is also a risk of inflating Type 1 error. Our multiple usage of simple tests such as chi-squared tests were deliberate, and unadjusted findings can point to avenues in need of future research. Drug use disorder was the only psychiatric disorder that was more common among persons with AUD compared to those without, which was the reason why we opted to include drug use disorder in the regression model. The number of variables that could be included in the model was limited by sample size, which meant that we could not address issues of other types of dual diagnoses. We did include the obvious confounders age, sex and AUDIT score to provide a measurement of AUD severity, but there are likely to be other types of confounding that were not addressed in our study. We were unable to compare characteristics of participants and non-participants. The European General Data Protection Regulation law (GDPR) and our ethics board permission prohibited any data collection related to patients who declined participation in the study. Thus, reasons for declining participation could not be recorded. However, the participation rate was high in the present study considering patients were recruited in a very sensitive situation immediately following an episode of self-harm. Since this study was conducted in Sweden, the findings may not directly translate to other cultural environments where suicidal behavior might be managed differently. Investigating the relationship between AUD and repeat suicidal behavior across other geographic and cultural settings could be an important path for future research.

### Implications

The fact that two thirds of persons with AUD in our study were only detected using the MINI may suggest a tendency towards clinical underdiagnosing of this disorder. Since clinical diagnoses were retrieved from patient psychiatric records at the time of the index episode, it might reflect a psychiatric emergency care problem where some diagnoses may be regarded as less relevant than others in the context of suicidal behavior. If the clinician’s focus is on symptoms of depression as well as suicide risk assessment, substance use issues might be missed, especially if the self-harm episode did not involve alcohol or if the patient was not previously diagnosed with AUD. Improved recognition of alcohol use disorder as well as changes in drinking patterns may aid clinicians in the assessment and management of patients who self-harm. Correct diagnostics are relevant not only for meeting the treatment needs of persons who present at psychiatric emergency departments in connection with self-harm, but also for the development for targeted public health initiatives to reduce suicidal behavior. The relatively low rate of recognition of AUD in suicide attempters also has relevance for register-based suicide research, as many persons with AUD will be incorrectly categorized if diagnoses are based solely on diagnostic codes.

### Conclusion

Clinicians need to consider the possibility of problematic alcohol use when evaluating patients who present with self-harm. Our findings suggest that the short-term risk of subsequent suicidal behavior may be elevated in patients with AUD. Educational interventions to improve recognition of alcohol use disorder may aid clinicians in the assessment and care of patients who present with self-harm.

## Data availability statement

The original contributions presented in the study are included in the article/**Supplementary material**. Further inquiries can be directed to the corresponding author.

## Ethics statement

The studies involving humans were approved by The Regional Ethics Committee in Gothenburg (589-10, T034-12). The studies were conducted in accordance with the local legislation and institutional requirements. The participants provided their written informed consent to participate in this study.

## Author contributions

PO: Formal analysis, Investigation, Methodology, Software, Validation, Writing – original draft, Writing – review & editing. SW: Data curation, Investigation, Validation, Writing – original draft, Writing – review & editing. LS: Formal analysis, Investigation, Methodology, Supervision, Validation, Writing – original draft, Writing – review & editing. ER: Conceptualization, Funding acquisition, Investigation, Validation, Writing – review & editing. BR: Conceptualization, Funding acquisition, Investigation, Validation, Writing – original draft, Writing – review & editing. MW: Conceptualization, Funding acquisition, Investigation, Supervision, Validation, Writing – original draft, Writing – review & editing.

## References

[1] Fazel S Runeson B. Suicide. N Engl J Med. (2020) 382:266–74. doi: 10.1056/NEJMra1902944 PMC7116087 31940700

[2] Darvishi N Farhadi M Haghtalab T Poorolajal J. Alcohol-related risk of suicidal ideation, suicide attempt, and completed suicide: a meta-analysis. PloS One. (2015) 10:e0126870. doi: 10.1371/journal.pone.0126870 25993344 PMC4439031

[3] Ritchie H Roser M. Alcohol consumption. Our world in data . Available online at: https://ourworldindata.org/alcohol-consumption.

[4] Castillo-Carniglia A Keyes KM Hasin DS Cerdá M. Psychiatric comorbidities in alcohol use disorder. Lancet Psychiatry. (2019) 6:1068–80. doi: 10.1016/S2215-0366(19)30222-6 PMC7006178 31630984

[5] Isaacs JY Smith MM Sherry SB Seno M Moore ML Stewart SH. Alcohol use and death by suicide: A meta-analysis of 33 studies. Suicide Life Threat Behav. (2022) 52:600–14. doi: 10.1111/sltb.12846 35181905

[6] Conner KR Duberstein PR. Predisposing and precipitating factors for suicide among alcoholics: empirical review and conceptual integration. Alcohol Clin Exp Res. (2004) 28:6S–17S. doi: 10.1097/01.ALC.0000127410.84505.2A 15166632

[7] Hesselbrock M Hesselbrock V Syzmanski K Weidenman M. Suicide attempts and alcoholism. J Stud Alcohol. (1988) 49:436–42. doi: 10.15288/jsa.1988.49.436 3216647

[8] Koller G Preuss UW Bottlender M Wenzel K Soyka M. Impulsivity and aggression as predictors of suicide attempts in alcoholics. Eur Arch Psychiatry Clin Neurosci. (2002) 252:155–60. doi: 10.1007/s00406-002-0362-9 12242575

[9] Wojnar M Ilgen MA Czyz E Strobbe S Klimkiewicz A Jakubczyk A. Impulsive and non-impulsive suicide attempts in patients treated for alcohol dependence. J Affect Disord. (2009) 115:131–9. doi: 10.1016/j.jad.2008.09.001 PMC2702673 18835498

[10] Ledden S Moran P Osborn D Pitman A. Alcohol use and its association with suicide attempt, suicidal thoughts and non-suicidal self-harm in two successive, nationally representative English household samples. BJPsych Open. (2022) 8:e192. doi: 10.1192/bjo.2022.594 36325650 PMC9634588

[11] Kessler RC Borges G Walters EE. Prevalence of and risk factors for lifetime suicide attempts in the National Comorbidity Survey. Arch Gen Psychiatry. (1999) 56:617–26. doi: 10.1001/archpsyc.56.7.617 10401507

[12] Morin J Wiktorsson S Marlow T Olesen PJ Skoog I Waern M. Alcohol use disorder in elderly suicide attempters: a comparison study. Am J Geriatr Psychiatry. (2013) 21:196–203. doi: 10.1016/j.jagp.2012.10.020 23343493

[13] Haw C Hawton K Houston K Townsend E. Psychiatric and personality disorders in deliberate self-harm patients. Br J Psychiatry. (2001) 178:48–54. doi: 10.1192/bjp.178.1.48 11136210

[14] Hawton K Fagg J McKeown SP. Alcoholism, alcohol and attempted suicide. Alcohol Alcohol. (1989) 24:3–9. doi: 10.1093/oxfordjournals.alcalc.a044864 2920070

[15] Ness J Hawton K Bergen H Cooper J Steeg S Kapur N. Alcohol use and misuse, self-harm and subsequent mortality: an epidemiological and longitudinal study from the multicentre study of self-harm in England. Emerg Med J. (2015) 32:793–9. doi: 10.1136/emermed-2013-202753 25564479

[16] Haw C Houston K Townsend E Hawton K. Deliberate self-harm patients with alcohol disorders: characteristics, treatment, and outcome. Crisis. (2001) 22:93. doi: 10.1027//0227-5910.22.3.93 11831606

[17] Rossow I Amundsen A. Alcohol abuse and suicide: A 40-year prospective study of Norwegian conscripts. Addiction. (1995) 90:685–91. doi: 10.1111/j.1360-0443.1995.tb02206.x 7795504

[18] Berglund M. Suicide in alcoholism: A prospective study of 88 suicides: I. The multidimensional diagnosis at first admission. Arch Gen Psychiatry. (1984) 41:888–91. doi: 10.1001/archpsyc.1984.01790200070009 6466048

[19] Beck AT Steer RA Trexler LD. Alcohol abuse and eventual suicide: a 5-to 10-year prospective study of alcohol-abusing suicide attempters. J Stud Alcohol. (1989) 50:202–9. doi: 10.15288/jsa.1989.50.202 2724967

[20] Sinclair JM Hawton K Gray A. Six year follow-up of a clinical sample of self-harm patients. J Affect Disord. (2010) 121:247–52. doi: 10.1016/j.jad.2009.05.027 19564047

[21] Riedi G Mathur A Seguin M Bousquet B Czapla P Charpentier S. Alcohol and repeated deliberate self-harm. Crisis [Internet]. (2012) 33(6):358–63. doi: 10.1027/0227-5910/a000148 22759664

[22] Bøe AS Mehlum L Melle I Qin P. Psychiatric disorders among adult deliberate self-harm patients and subsequent risk of dying by suicide, mental and behavioural disorders and other external causes. J Psychiatr Res. (2023) 165:83–90. doi: 10.1016/j.jpsychires.2023.07.011 37481790

[23] Bolton JM Gunnell D Turecki G. Suicide risk assessment and intervention in people with mental illness. BMJ. (2015) 351:h4978. doi: 10.1136/bmj.h4978 26552947

[24] Lindh ÅU Waern M Beckman K Renberg ES Dahlin M Runeson B. Short term risk of non-fatal and fatal suicidal behaviours: the predictive validity of the Columbia-Suicide Severity Rating Scale in a Swedish adult psychiatric population with a recent episode of self-harm. BMC Psychiatry. (2018) 18:319. doi: 10.1186/s12888-018-1883-8 30285661 PMC6167823

[25] Lindh ÅU Dahlin M Beckman K Strömsten L Jokinen J Wiktorsson S. A comparison of suicide risk scales in predicting repeat suicide attempt and suicide: a clinical cohort study. J Clin Psychiatry. (2019) 80:0–. doi: 10.4088/JCP.18m12707 31747488

[26] Olsson P Wiktorsson S Strömsten LM Salander Renberg E Runeson B Waern M. Attention deficit hyperactivity disorder in adults who present with self-harm: a comparative 6-month follow-up study. BMC Psychiatry. (2022) 22:1–9. doi: 10.1186/s12888-022-04057-0 35751076 PMC9233312

[27] Posner K Brown GK Stanley B Brent DA Yershova KV Oquendo MA. The Columbia–Suicide Severity Rating Scale: initial validity and internal consistency findings from three multisite studies with adolescents and adults. Am J Psychiatry. (2011) 168:1266–77. doi: 10.1176/appi.ajp.2011.10111704 PMC3893686 22193671

[28] Sheehan DV Lecrubier Y Sheehan KH Amorim P Janavs J Weiller E. The Mini-International Neuropsychiatric Interview (MINI): the development and validation of a structured diagnostic psychiatric interview for DSM-IV and ICD-10. J Clin Psychiatry. (1998) 59:22–33. 9881538

[29] Stanley B Traskman-Bendz L Stanley M. The suicide assessment scale: a scale evaluating change in suicidal behavior. Psychopharmacol Bull. (1986) 22:200–5. 3726067

[30] Harriss L Hawton K. Suicidal intent in deliberate self-harm and the risk of suicide: the predictive power of the Suicide Intent Scale. J Affect Disord. (2005) 86:225–33. doi: 10.1016/j.jad.2005.02.009 15935242

[31] Saunders JB Aasland OG Babor TF de la Fuente JR Grant M. Development of the alcohol use disorders identification test (AUDIT): WHO collaborative project on early detection of persons with harmful alcohol consumption-II. Addiction. (1993) 88:791–804. doi: 10.1111/j.1360-0443.1993.tb02093.x 8329970

[32] WHO. AUDIT: The alcohol use disorders identification test: Guidelines for use in primary health care. Geneva, Switzerland: World Health Organization (2001).

[33] Maurex L Lekander M Nilsonne Å Andersson EE Åsberg M Öhman A. Social problem solving, autobiographical memory, trauma, and depression in women with borderline personality disorder and a history of suicide attempts. Br J Clin Psychol. (2010) 49:327–42. doi: 10.1348/014466509X454831 19555523

[34] Svanborg P Åsberg M. A new self-rating scale for depression and anxiety states based on the Comprehensive Psychopathological Rating Scale. Acta Psychiatr Scand. (1994) 89:21–8. doi: 10.1111/j.1600-0447.1994.tb01480.x 8140903

[35] Tyrer P Owen R Cicchetti D. The brief scale for anxiety: a subdivision of the comprehensive psychopathological rating scale. J Neurol Neurosurg Psychiatry. (1984) 47:970–5. doi: 10.1136/jnnp.47.9.970 PMC1028000 6481391

[36] Scheffer J. Dealing with missing data. Res Lett Inf Math Sci. (2002) 3:153–60.

[37] Murphy GE Robins E. Social factors in suicide. JAMA. (1967) 199:303–8. doi: 10.1001/jama.199.5.303 6071132

[38] Berglund M Ojehagen A. The influence of alcohol drinking and alcohol use disorders on psychiatric disorders and suicidal behavior. Alcohol Clin Exp Res. (1998) 22:333s–45s. doi: 10.1097/00000374-199807001-00010 9799958

[39] Borges G Walters EE Kessler RC. Associations of substance use, abuse, and dependence with subsequent suicidal behavior. Am J Epidemiol. (2000) 151:781–9. doi: 10.1093/oxfordjournals.aje.a010278 10965975

[40] Frances RJ Franklin J Flavin DK. Suicide and alcoholism. Am J Drug Alcohol Abuse. (1987) 13:327–41. doi: 10.3109/00952998709001517 3687894

[41] Sher L. Alcohol consumption and suicide. QJM. (2005) 99:57–61. doi: 10.1093/qjmed/hci146 16287907

[42] Lundholm L Thiblin I Runeson B Leifman A Fugelstad A. Acute influence of alcohol, THC or central stimulants on violent suicide: A Swedish population study. J Forensic Sci. (2014) 59:436–40. doi: 10.1111/1556-4029.12353 24745078

[43] Case A Deaton A. Rising morbidity and mortality in midlife among white non-Hispanic Americans in the 21st century. Proc Natl Acad Sci U S A. (2015) 112:15078–83. doi: 10.1073/pnas.1518393112 PMC4679063 26575631

[44] Copeland WE Gaydosh L Hill SN Godwin J Harris KM Costello EJ. Associations of despair with suicidality and substance misuse among young adults. JAMA Netw Open. (2020) 3:e208627–e. doi: 10.1001/jamanetworkopen.2020.8627 PMC7312388 32573708

[45] Sterling P Platt ML. Why deaths of despair are increasing in the US and not other industrial nations—insights from neuroscience and anthropology. JAMA Psychiatry. (2022) 79:368–74. doi: 10.1001/jamapsychiatry.2021.4209 35107578

[46] Polydorou S Gunderson EW Levin FR. Training physicians to treat substance use disorders. Curr Psychiatry Rep. (2008) 10:399–404. doi: 10.1007/s11920-008-0064-8 18803913 PMC2741399

[47] Madsen T Erlangsen A Hjorthøj C Nordentoft M. High suicide rates during psychiatric inpatient stay and shortly after discharge. Acta Psychiatr Scand. (2020) 142:355–65. doi: 10.1111/acps.13221 32715465

