# Supplementary material for: Clinical characteristics and 6-month follow-up of adults with and without alcohol use disorder who self-harm
Source: Front Psychiatry. 2024 Aug 2;15:1396855. doi: 10.3389/fpsyt.2024.1396855 (PMC11327149; doi:10.3389/fpsyt.2024.1396855)
Supplement: Supplementary file 1 [file Table_1.docx]

|  | **Index episode** | | **Follow-up** |
| --- | --- | --- | --- |
|  | **SA, *n* = 666** | **NSSI, *n* = 138** | **Fatal and non-fatal suicidal behavior**^a^**, *n* = 165** |
| **Method** | *n* (%) | | *n* (%) |
| Poisoning | 470 (70.6) | 37 (26.8) | 126 (76.4) |
| Gassing | 6 (0.9) | - | - |
| Hanging/strangulation/choking | 78 (11.7) | 5 (3.6) | 26 (15.8) |
| Drowning | 15 (2.3) | - | 2 (1.2) |
| Cutting | 117 (17.6) | 95 (68.8) | 27 (16.4) |
| Jumping | 23 (3.5) | - | - |
| Vehicular | 26 (3.9) | 1 (0.7) | 3 (1.8) |
| Firearm/explosives | 3 (0.5) | - | - |
| Other method | 18 (2.7) | 21 (15.2) | 7 (4.2) |

**Supplementary Table S1.** Methods employed in connection with suicide attempt (SA)/non-suicidal self-injury (NSSI).

SA Suicide attempt, NSSI Non-suicidal self-injury
 ^a^ Includes 10 suicides.

| **Supplementary Table S2.** Logistic regression models. | | | | | | | | | | | | | | |
| --- | --- | --- | --- | --- | --- | --- | --- | --- | --- | --- | --- | --- | --- | --- |
|  | **Model** | | | | |  | **Predictor** | | | | | | | |
|  | **Nagelkerke *R^2^*** | ***χ^2^*** | | ***P*** | |  | | **B** | **SE** | **Wald** | ***df*** | ***P*** | **Exp(B)** | **95% CI** |
|  |  |  | |  | |  | |  |  |  |  |  |  |  |
| **Prediction of reported increase in alcohol consumption prior to index** | | |  | |  | | | |  |  |  |  |  |  |
| AUD + sex + age | ..22 | 113.74 | | <.001 | |  | |  |  |  |  |  |  |  |
| AUD |  |  | |  | |  | | 1.97 | 0.20 | 93.00 | 1 | <.001 | 7.16 | 4.80-10.69 |
| Sex |  |  | |  | |  | | -0.14 | 0.21 | 0.43 | 1 | .51 | 0.87 | 0.58-1.32 |
| Age |  |  | |  | |  | | -0.01 | 0.01 | 3.34 | 1 | .07 | 0.99 | 0.98-1.00 |
| **Prediction of reported relationship problems prior to index** | | | | | | | | | | | | | | |
| AUD + sex + age | .08 | 45.37 | | <.001 | |  | |  |  |  |  |  |  |  |
| AUD |  |  | |  | |  | | 0.43 | 0.17 | 6.43 | 1 | .01 | 1.54 | 1.10-2.15 |
| Sex |  |  | |  | |  | | 0.49 | 0.16 | 9.30 | 1 | .002 | 1.64 | 1.19-2.25 |
| Age |  |  | |  | |  | | -0.02 | 0.00 | 23.50 | 1 | <.001 | 0.98 | 0.97-0.99 |

AUD Alcohol Use Disorder
