# Supplementary material for: Clinical characteristics and 6-month follow-up of adults with and without alcohol use disorder who self-harm
Source: Front Psychiatry. 2024 Aug 2;15:1396855. doi: 10.3389/fpsyt.2024.1396855 (PMC11327149; doi:10.3389/fpsyt.2024.1396855)
Supplement: Supplementary file 2 [file Table_2.docx]

| **Supplementary Table S3.** Instruments and scale items used in the study, ratings and total score ranges | | | |
| --- | --- | --- | --- |
| **Instrument** | **What it measures** | **Items used in this study** | **Rating** |
| **AUDIT, Alcohol Use Disorders Identification Test** | Alcohol consumption,  consumption patterns, symptoms of dependence and harmful use, and consequences of alcohol use | All items (total score) | Ten questions scored 0-4 (total score range 0-40) |
| **Borderline Symptom Scale (self-rated, subscale of KABOSS, Karolinska Affective and Borderline Symptoms Scale)** | Symptoms associated with emotionally unstable personality disorder | All items (total score) | Eight items scored 0-6 (total score range (0-48) |
|  |  | Item rating feelings of abandonment | **0** I rarely feel abandoned **1** -  **2** Sometimes I can feel abandoned.  **3** -  **4** I very often feel abandoned.  **5** -  **6** I have difficult and painful feelings almost all the time of abandonment or loneliness.  A rating >2 was used to denote feelings of abandonment. |
| **BSA, Brief Scale for Anxiety (self-rated, subscale of KABOSS)** | Symptoms of anxiety | All items (total score) | Nine items scored 0-6 (total score range 0-54) |
| **C-SSRS, Columbia Suicide Severity Rating Scale** | History and characterization of self-harm behavior | Item 12b: Actual suicide attempt (at index episode) | Yes/no  Self-harm behavior lacking suicidal intent was classified as non-suicidal self-injury (NSSI). |
|  |  | Item 21a: Medical lethality (index attempt) | **0** None or very minor physical harm  **1** Minor physical harm  **2** Moderate physical injury  **3** Moderately severe physical injury  **4** Severe physical injury (Intensive care required)  **5** Death  A rating ≥3 was used to denote high medical lethality |
| **MADRS-S, Montgomery-Åsberg Depression Rating Scale-self-rated (subscale of KABOSS)** | Symptoms of depression | All items (total score) | Nine items scored 0-6  (total score range 0-54) |
| **MINI, Mini Neuropsychiatric Interview** | Diagnostic criteria associated with common psychiatric disorders according to the DSM-5 | Fulfils diagnostic criteria for alcohol dependence (past 12 months) | Yes/no |
|  |  | Fulfils diagnostic criteria for alcohol abuse (past 12 months) | Yes/no |
| **Suicide Assessment Scale** | Observed and reported symptoms that associate with suicide risk | Item 11: Trait impulsivity | **0** Satisfies their needs thoughtfully and with consideration for possible consequences.  **1** May occasionally satisfy impulsive needs without considering the consequences.  **2** Often has difficulty controlling their impulsive needs and urges, but knowledge of the consequences is limiting.  **3** Mostly satisfies their impulsive needs and impulses without any thought of the consequences.  A rating ≥2 was used to denote trait impulsivity. |
|  |  | Item 13: Hopelessness | **0** Plans for and believes in the future.  **1** Can temporarily see the future darkly and pessimistically.  **2** Often sees the future darkly and pessimistically. Hopeful thoughts rare.  **3** Looking at the future only with dark and pessimistic forebodings. Never sees any light at the end of the tunnel.  **4** Is completely resigned, completely convinced that the absolute worst will happen.  A rating ≥2 was used to denote feelings of hopelessness. |
|  |  | Item 12: Worthlessness | **0** Normal, adequate belief in one's own ability.  **1** Can temporarily lose faith in one's own abilities, but can easily correct oneself.  **2** Usually normal self-confidence, but periods of failure and failing faith in one's own ability.  **3** Often difficult to correct feeling of worthlessness and failure, strong self-doubt. The own self-confidence very doubtful.  **4** Psychotic experience of worthlessness and failure.  A rating ≥2 was used to denote feelings of worthlessness. |
|  |  | Item 9: Worries/anxiety | **0** No abnormal worry or anxiety.  **1** Easier than usual to get worried.  **2** Gets anxious and worried easily, exaggerates worries, but the quiet moments prevail.  **3** Is rarely or never calm, worry and fear of the present and the future take over.  **4** Constantly has severe anxiety and severe feelings of uneasiness; tormented by worry and fear.  A rating ≥2 was used to denote worries/anxiety. |
| **Suicide Intent Scale** | Level of suicidal intent in connection with a suicidal act | Item 15: Impulsivity in connection with the index attempt | **0** No preparations (impulsive act)  **1** Deliberated for 3 hours or less before act  **2** Deliberated for more than 3 hours before act  A rating of 0 was used to denote impulsivity in connection with the index attempt. |
